# Gender Differences in Oral Health: Self-Reported Attitudes, Values, Behaviours and Literacy among Romanian Adults

**DOI:** 10.3390/jpm12101603

**Published:** 2022-09-29

**Authors:** Ruxandra Sfeatcu, Beatrice Adriana Balgiu, Christina Mihai, Ana Petre, Mihaela Pantea, Laura Tribus

**Affiliations:** 1 Department of Oral Health and Community Dentistry, Faculty of Dental Medicine, “Carol Davila” University of Medicine and Pharmacy, 17–21 Calea Plevnei Street, 010221 Bucharest, Romania; 2 Department of Career and Educational Training, University Politehnica of Bucharest, 313 Splaiul Independenţei, 060042 Bucharest, Romania; 3 Department of Preventive Dentistry, Faculty of Dental Medicine, “Carol Davila” University of Medicine and Pharmacy, 17–21 Calea Plevnei Street, 010221 Bucharest, Romania; 4 Department of Aesthetics in Dental Medicine, Faculty of Dental Medicine, “Carol Davila” University of Medicine and Pharmacy, 17–21 Calea Plevnei Street, 010221 Bucharest, Romania; 5 Department of Fixed Prosthodontics and Occlusology, Faculty of Dental Medicine, “Carol Davila” University of Medicine and Pharmacy, 17–23 Calea Plevnei Street, 010221 Bucharest, Romania; 6 Department of Internal Medicine, Faculty of Dental Medicine, “Carol Davila” University of Medicine and Pharmacy, 17–21 Calea Plevnei Street, 010221 Bucharest, Romania

**Keywords:** attitudes, values, behaviour, gender differences, oral health literacy

## Abstract

A topic that has been less researched on, especially in Romania, is the identification of gender differences in oral health. The present scientific research used an online survey to assess the attitudes (dental beliefs survey-R/R-DBS), the values (oral health values scale/OHVS), the behaviours (dental neglect scale/DNS), and the literacy (oral health literacy scale/OHLS) related to oral health and to dental professional services in the case of a sample of 600 Romanian adults (Mean_age_ = 30.84). The relation between the level of knowledge as a predictor of attitudes and values related to oral health was analysed by the means of a structural equation based on the partial least square method (PLS-SEM). The results show that women have more positive attitudes towards dental medical services, higher oral health values, better self-care behaviours, and higher oral health literacy than men. In the case of men, attitudes and behaviours related to self-care are influenced by their oral health literacy and level of education; in the case of women, the latter are influenced only by their level of oral health literacy. The impact that knowledge in oral health has on attitudes, values, and behaviours was highlighted. The differences in attitudes and values related to oral health between the two genders can be accounted for by the differences in formal and informal education (studies and oral health literacy, respectively).

## 1. Introduction

The scientific dental literature indicates that perceptions, attitudes, beliefs, and individual values generate the state of one’s oral health [1,2,3]. Thus, individuals with good oral health are defined by high quality attitudes and values, as well as by positive behaviours related to medical oral care [2,4,5,6].

Given that studies found that women’s oral health is better than men’s [7,8,9,10] and that women resort to dental services more [11,12], it is very likely that there are different perceptions, attitudes, and behaviours underlying these differences.

Although several studies that approached gender differences related to one’s state of health [7,8,13,14], empirical findings on the values, mainly, and attitudes that differentiate gender psycho-sociology specific to oral health care are relatively few [9,15].

Unlike other countries which use national databases to explore the effect of gender on oral health and hygiene behaviours [8,16], in the Romanian scientific literature, gender differences related to oral health are only incidentally approached in studies on oral health. Since we did not find any Romanian scientific studies on the differences in attitudes, values, and behaviours regarding oral health corresponding to each gender, the purpose of this study was to assess gender psychology in oral health care, in the case of a sample of Romanian adults.

### 1.1. Gender Differences Regarding Trust in Dental Services

In the specialized literature, there is a consensus which shows that women are more interested in oral health, which leads to trust in dental services [12,14,17]. Women have more favourable, more precise, and more stable convictions related to dental professional services [6] and, consequently, better oral hygiene, less periodontal diseases, less frequent loss of teeth caused by cavities, and better self-assessed oral health [6,17]. This is noticeable from adolescence when girls tend to have clearer and healthier beliefs when it comes to oral health and general health when compared to boys [2]. Other studies show that women resort to dental services more often [11] and are more likely to follow medical instructions and to pay dental visits, in comparison to males [17].

### 1.2. Evidence for Gender Differences in Oral Health Values

Oral health values have been less studied in the literature; they refer to the importance that an individual gives to the preservation of dental health and care by adding auxiliary hygiene methods to tooth brushing [18]. Studies show that, as long as individuals care about oral health, they think favourably of dentists and the oral healthcare systems [18,19]. For example, these values make people resort to professional dental services [20] and take appropriate care of their daily oral hygiene [18].

Studies have shown that women are more interested in oral health and hygiene than men [17,21]; women value the usage of dental floss more than men (*p* < 0.001) [17]. There is not always a consensus of research. Certain research findings demonstrated that both men and women have almost the same level of knowledge on oral health, but they have different interests and behaviours; for example, women are more interested in their appearance and beauty than men [17]. Other studies show that good oral health of women has derived from good oral health knowledge [9]. Previously mentioned assumptions can be explained by the fact that women are generally more concerned with their dental aesthetics [8] and value their smile [22]. The most frequently used methods of taking care of oral health are tooth brushing [17,23] and flossing [16,18,24]. In addition, women have other standards related to facial aesthetics that influence success in one’s activity and social interactions [25].

### 1.3. Gender Differences Regarding Behaviours Related to Oral Health

The most relevant studies show that women’s behaviour related to routine oral hygiene is highly superior [10,13,26]. Brushing and flossing are significantly frequent in women [17]. Women have better results than men when it comes to activities regarding oral health: the high frequency of brushing, a better basic approach of oral hygiene, flossing, and more frequent visits to the dentist [10,27].

Generally, men use medical assistance services less frequently and they are less likely to ask for preventive care [9,12,17,26,28]. Men’s less healthy behaviours are due to poorer knowledge in the domain of oral health and a weak positive attitude regarding oral health [9,13]. Men are more likely to ignore oral health, to have poor oral hygiene habits, and to experiment higher rates of periodontal diseases, mouth cancer, and dental trauma. In addition, in comparison with women, men go to the dentist mainly when they have an acute problem, but not necessarily for the prevention of oral diseases [8,9].

### 1.4. Explanations Regarding Gender Differences in Oral Health

Various explanations were given in the analyses of differences in attitudes and behaviours related to oral health. Most of the studies conclude that women can have better oral health due to their higher knowledge on oral health [9]. In their turn, knowledge on oral health leads to a greater awareness of the importance of oral health and to better oral health [17,29]. Research on oral health demonstrated the KAP (knowledge, attitudes, practice) theory which underlines the positive relationship between knowledge and practice, mediated by attitudes [3]. In addition, more visits to the dentist result in more opportunities to get educated and informed on health problems [30]. As for oral health literacy, studies found contradictory data: they either demonstrated that men and women have a relatively equal level of information and knowledge [17], or young women have much higher levels in comparison with men of the same age [31], while in the case of older individuals, men have higher levels of knowledge, which are associated with life quality [32]. Other explanations are related to the perception of a good health condition, in the case of men. Diseases are mostly associated with the loss of masculinity [8]; therefore, it is not surprising that men use dental services less frequently than women. In exchange, women believe that oral health has a bigger impact on their aspect and well-being [33]. This was predictable, given that women give more importance to oral health and prevention, and they are more likely to visit the dentist and to look for preventive care. In addition, it is reported that women, in comparison with men, have a lower self-assessment regarding oral health [30], and therefore, they tend to be more prepared to adopt a better behaviour towards oral health as they continue to gather knowledge on dental health [15].

## 2. Materials and Methods

### 2.1. Data Collection

The present study was based on a cross-sectional design through which the data collection was made between October and November 2021 by the means of an online survey. The latter was distributed on the most known social media networks, such as Facebook and WhatsApp and email addresses, using the snowball method. Thus, the sampling strategy was one of convenience. The eligibility conditions of the participants were: 18 years of age and Romanian residency. The questionnaire was secured to be completed only once by every participant. The platform built by authors only recorded the biological sex and the only existing options were “male” and “female”. It took participants around 10–12 min to fill in the questionnaires. In the first part of the form, the informed consent for the participants was included, the objective of the study was explained, and the anonymity of the answers was ensured, in accordance with GDPR rules and regulations; the participants were also informed that they can renounce at any time with no other consequences. The participation in the poll was voluntary and it was not rewarded. 

### 2.2. Ethical Consideration

The study was conducted in full accordance with ethical principles, including the World Medical Association Declaration of Helsinki from 1975, as revised in 2013. The study was approved by the Scientific Research Ethics Committee of “Carol Davila” University of Medicine and Pharmacy, Bucharest, Romania (Protocol No. 28447/18.10.2021). 

### 2.3. Measures

**The dental beliefs survey-R/R-DBS** [34,35,36] measures patient attitudes regarding their relationship with the dentist and the dental services. In this study, we used the 28-item version, assessed on a 5-point Likert-type scale from 1—*never* to 5—*nearly always*, distributed in three subscales: professionalism, comfort, and lack of control [36]. Studies have shown good internal consistency: 0.95 in the case of college students [36]. In addition, the instrument validated on a sample of Romanian adults showed good psychometric properties [37]. In the case of the present study, the Cronbach’s α coefficient for the total score = 0.96 (95% CI—0.95–0.96), McDonald’s ω = 0.96 (95% CI—0.96–0.97), and CFA showed the following coefficients: χ^2^/df = 3.79; CFI = 0.93; TLI = 0.92; RMSEA = 0.068 (90% CI—0.064–0.078); SRMR = 0.044.

**The oral health values scale/OHVS** [18] measures the relevant domains of the values regarding oral health. The scale is made of 12 items assessed on a scale from 1—*strongly disagree* to 5—*strongly agree* and it contains 4 subscales, each with 3 items: professional dental care, appearance and health, flossing, and retention of natural teeth. The total score of the scale is between 12 and 60. The scale validated on the general Romanian population showed good psychometric properties [24]. In this study, internal consistency coefficients of Cronbach’s α = 0.77 (95% CI—0.75–0.80), McDonald’s ω = 0.78 (95% CI—0.75–0.80), while CFA showed the following coefficients: χ^2^/df = 4.08; CFI = 0.97; TLI = 0.96; RMSEA = 0.042 (90% CI—0.030–0.054); SRMR = 0.036.

**The dental neglect scale/DNS** [13,38] is used in order to measure adult behaviours and attitudes involved in oral self-care. The scale contains 6 items (one is reverse—[36] Coolidge et al., 2009), assessed on a continuum from 1—*strongly disagree* to 5—s*trongly agree*, and it is unifactorial. The Cronbach’α coefficient reported by the authors of the instrument was 0.71 [38]. The scale, recently validated on the Romanian adult population, demonstrated good psychometric properties [39]. In the present study, Cronbach’s α was 0.71 (95% CI—0.67–0.75), McDonald’s ω = 0.71 (95% CI—0.67–0.74); in terms of CFA, a good fit was obtained: χ^2^/df = 2.46; CFI = 0.98; TLI = 0.97; RMSEA = 0.049 (90% CI—0.022–0.078); SRMR = 0.026. 

**The oral health literacy scale/OHLS** was assessed through three questions adapted from the literature [40]; these questions check the understanding of the medical information. Sample item: *How certain are you of the fact that you can fill in the personal data from your medical record, informed consent, and the evaluation form of your general health state?* The items were self-assessed on a continuum from 1—*not at all* to 5—*very certain*. The reliability of the newly created scale was acceptable: Cronbach’s α was 0.63 (95% CI—0.55–0.65) and McDonald’s ω = 0.62 (95% CI—0.55–0.66).

### 2.4. Socio-Demographic Data

The collected sociodemographic data took into consideration gender (male, female), age, studies (primary, secondary, university, post-university studies), residency (urban vs. rural), the working sector (public, private, other), and geographical region (the eight main regions of the country were included).

### 2.5. Hypotheses

1. Female subjects have better superior attitudes and values regarding oral health, a higher trust in dental service professionalism, greater literacy regarding oral health, and consequently, better self-care behaviours in comparison to the male subjects in the sample.
2. The difference in attitudes, values, and behaviours of oral health is influenced by the individual level of knowledge given by education and oral health literacy (OHL).

### 2.6. Data Analysis

In view of analysing the data, the normality assumptions were examined. Thus, the data normality condition was verified by calculating the skewness and kurtosis indicators and by using the Shapiro–Wilk test (*p* < 0.001). In order to compare the groups according to gender, the Mann–Whitney U non-parametric test was used. The statistically significant differences were considered to be at *p* < 0.001. The relationship between the values, attitudes, and behaviours of the two groups of males and females, on the one hand, and the knowledge level (made of the educational level and the oral health literacy) were analysed through structural equation modelling using the partial least square technique (PLS-SEM), which has the advantage of robustness compared to the other models. Firstly, the models made on a male/female subsample were verified through reliability (this was conducted through Dijkstra-Henseler’s rho (ρA), Jöreskog’s rho (ρc), and Cronbach α coefficients—it is recommended that they are >0.70 [41], with convergence validity (average variance extracted (AVE)); AVE is considered to be good if it is >0.50 [42] with discriminat validity (the robust indicator, heterotrait monotrait ratio of correlations—HTMT—with a recommended cut-off level at <0.85)). The multicollinearity between variables was verified through the scores of the variance inflation factor (VIF), whose value must be <5 [43]. Secondly, we assessed the relations between latent constructs. The factorial structures of the research instruments were assessed using confirmatory factor analysis (CFA), and the reliability was established using the Cronbach α and the McDonald ω coefficients. All the data were analysed with programs SPSSv22 (IBM, New York, NY, USA) and ADANCO 2.3.1. (Composite Modeling GmbH & Co. KG, Kleve, Germany).

## 3. Results

### 3.1. The Socio-Demographic Characteristics of the Groups

The resulting sample consisted of 600 respondents with a Mean_age_ of 30.84 (S.D. = 14.34); the youngest respondent was 18 years old, and the oldest was 74 years old); the sample consisted of 350 female subjects (Mean_age_ = 29.51 +/− 14.47) and 250 male subjects (Mean_age_ = 31.79 +/− 14.11). The samples had relatively equal characteristics. *Regarding the female sample*: 44.80% worked in the private sector, 50.80% worked in the public sector, 4.40% did not work; 85.10% lived in the city and 14.90% in the countryside; 3% had primary studies, 16.10%—secondary studies, 57.22%—university studies and 23.68%—post-university. *Regarding the male sample*: 47.60% worked in the private sector, 44.60% worked in the public sector, 7.80 did not work; 84.50% lived in the city and 15.50% in the countryside; 4% had primary studies, 26.30%—high school, 47.00%—university studies and 22.70% had post-university studies (Table 1). 

### 3.2. Gender Differences

Table 2 shows the descriptive statistics (means, S.D., skewness and kurtosis and consistency coefficients). Since skewness and kurtosis values are higher than they should—1.00–1.00 [43]—and the Shapiro–Wilk test is statistically significant, the normality assumptions are considered to be violated. At the same time, the calculation of Z scores for skewness and kurtosis, as a ratio between the absolute value and the standard error, demonstrate that the latter are not in the interval (−3.29, 3.29), thus, corresponding to a normal distribution [44].

As a result, the gender difference was calculated by the means of the Mann–Whitney U coefficient (Table 3). The female respondents have significantly higher scores for all the scales (all at the *p* < 0.01). The data show that women invest time and energy in dental professional services (Z = −4.81), they pay attention to their appearance in public, and they are more interested in home personal care than men are (Z = −8.63). In addition to the brushing technique, women invest in flossing behaviours (Z = −7.05). At the same time, oral health literacy differentiates between the two genders (Z = −9.27). 

An in-depth analysis of the items helped us to find out the most important gender differences. Table 4 shows 12 items that cause the biggest differences between the two groups. The items with the smallest differences, located at the opposite extreme, are listed in the Appendix A Table A1.

It can be noticed that men are more likely to believe that dentists limit the access to information and they do not offer all the right information for the patient to be able to make the right decisions (Items 1–2). Men also believe that the medical staff can make patients feel guilty with regard to dental care (item 3). Out of the four items that differentiate males from females in terms of values, one can notice the reason why women invest more in oral health, namely, the need to capitalize on their facial appearance as a way of influencing self-image and interpersonal relations (items 6 and 8). Women have a better understanding of the medical information (Z= −5.58). 

### 3.3. Structural Equation Model

#### 3.3.1. Male Sample

**Figure 1:**
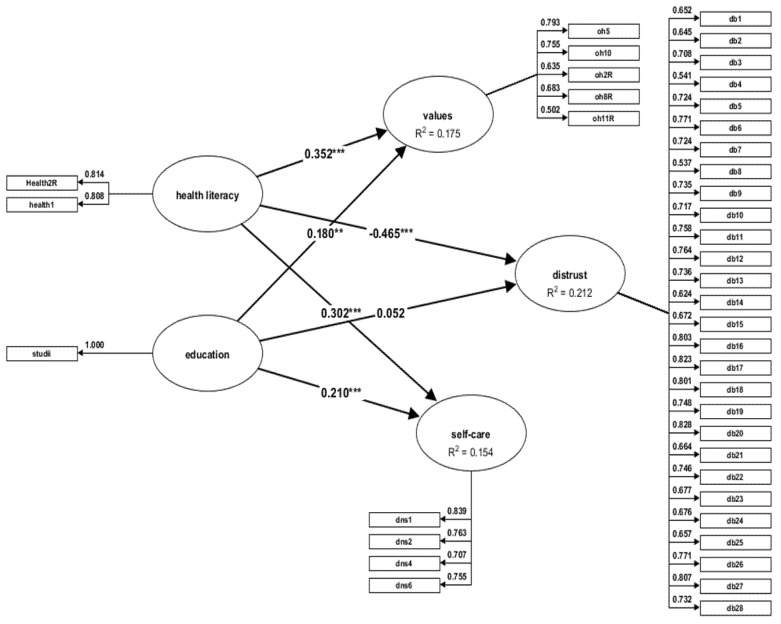
Structural equation model - male sample. ** *p* = 0.004; *** *p* = 0.000.

**Figure 2:**
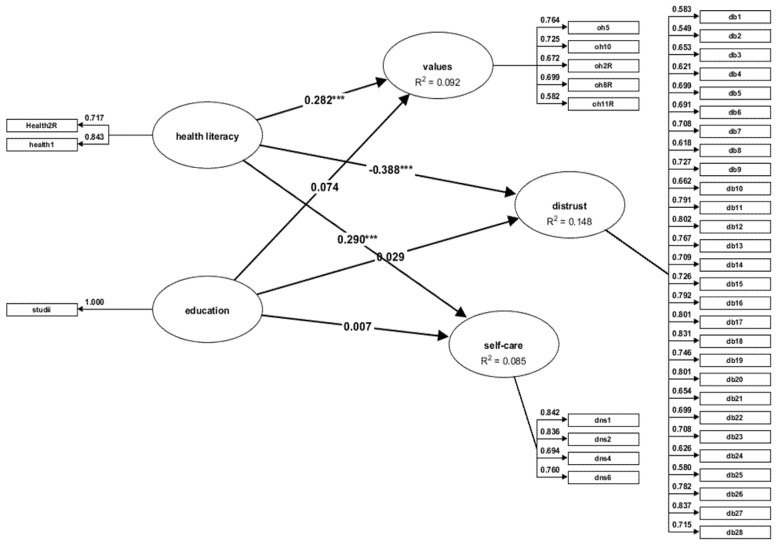
Structural equation model - female sample; *** *p* = 0.000.

We verified the relationship between the OHL level of knowledge and the education level and values, attitudes, and behaviours related to oral health. After we excluded the constructs for which the value of the coefficient was <0.50 (one item from OHLS, seven items from OHVS, and two items from DNS) [45], we created a reflective model which was verified for each of the two samples, males/females (Figure 1 and Figure 2, respectively). 

The assessment of the coefficients we mentioned (Dijkstra-Henseler’s rho—ρA, Jöreskog’s rho—ρc, and Cronbach α) shows that they have values which are >0.70, except for the value of rho—ρ_A_ for the oral health literacy construct (OHL), which is 0.48. The values of AVE for all the factors are >0.50, and only the value of the oral health construct is 0.46 (however, as long as the criterion of reliability is fulfilled, the low values of AVE are not considered to be problematic [46]. The variance inflation factor (VIF) has values between 1.13 and 4.38; therefore, there is no multicollinearity, apparently.

The values of HTMT are not >0.85, as they are between 0.24 and 0.71. Therefore, it is safe to say that the values of the indicators demonstrate the internal consistency of the model. Then, in order to assess the model, we made a bootstrap test with 5000 resamples, in accordance with Henseler’s recommendations [41] in order to generate the values of test *t* (*t*-test) and of the standard error of the parameters of the model. The correlations of the latent variables are significant if t > 1.96 and the level of value of is *p* < 0.05 [45]. Therefore, the analysis of the results of effects inference (Table 5) shows that both OHL and the educational level (EL) have a positive and significant impact on oral health values (β = 0.35; t = 6.22 and β = 0.18; t = 3.00, respectively) and on oral health self-care (β = 0.30; t = 5.04; and β = 0.21; t = 3.74, respectively). 

Only the low level of OHL influences the distrust of dentists (β = −0.46; t = −8.61) (*p* < 0.01), and EL has no impact on the attitudes and the beliefs regarding the dentist’s professionalism (β =0.05; t = 0.84) (*p* = 0.398). Figure 1 shows the path coefficients; OHL and the educational level account for 17% of the oral health value variance (R^2^ = 0.17) and 15% of the variance of the self-care behaviour (R^2^ = 0.15). The distrust of dentists is accounted for only by the low level of oral health literacy. 

#### 3.3.2. Female Sample

We used the same method of verification of the model characteristics in the case of the female sample. The values of the reliability coefficients are >0.70. The AVE has values that are >0.50 for all constructs, except for some low values in the case of the oral health value construct, which has a beta coefficient of 0.41 (a lower value for AVE does not make the model precarious, as long as the reliability criterion is met [46]. HTMT criteria in variance-based SEM is <0.85; the highest value is 0.77. The examination of multicollinearity shows that the variance inflation factor (VIF) has values between 1.61 and 4.80.

Oral health literacy (OHL) has a positive and significant impact on the values (β = 0.32; t = 7.50) and the self-care of oral health (β = 0.37; t = 6.54) and a negative impact on the distrust of dentists (β = −0.42; t = −10.38), while education (EL) has no significant effect on any of the dependent variables included (all at *p* < 0.01) (Table 6). Figure 2 shows the path coefficients for the associations between oral health literacy (OHL), the educational level (EL), self-care, and the distrust of dentists. 

OHL accounts for 9.2% of the oral health values (R^2^ = 0.09), 8% of the variance of the self-care behaviour (R^2^ = 0.08), and 14% of the variance of positive attitudes regarding dental services (R^2^ = 0.14).

## 4. Discussion and Conclusions

The topic of gender differences in oral health remains a productive domain in the study of oral health care. In order to reach an encompassing vision on gender psychology, the current study is centred on the analysis of the main pillars that make the difference between men and women regarding oral health—namely, attitudes, values, behaviours, and knowledge. The results of the research show significantly higher scores for the female subjects in comparison with male subjects regarding favourable attitudes towards dentists and medical cabinets, and a bigger investment in oral self-care. Women give more importance to flossing techniques and facial appearance, and they have higher oral health literacy. Men exhibit negative beliefs with regard to dentists, as they consider that dentists provide information that is not important enough and they disregard patient comfort. These aspects suggest that women prioritise oral health care as an integral part of the daily routine. The results corroborate prior studies which showed that female subjects have positive attitudes towards medical professional care [6,8,9,10,14], they invest more in oral care and auxiliary hygiene techniques such as flossing [17,18], and they have more oral knowledge on health than men [29].

The search of the literature empirically indicated that positive attitudes and behaviours for oral health are accounted for by one’s level of information [47]. We considered that a limited literacy ability among adults is one of the obstacles against better results in oral health, as other authors mentioned [9]. In this sense, the present study analysed the influence of OHL (oral health literacy) and education level (EL) on personal attitudes, values, and behaviours, within the two genders. In the case of both men and women, OHL (oral health literacy) influences the way in which dental care is perceived. In the case of males, formal education (studies)—besides OHL—has a significant contribution to the degree to which they invest in their own dental health. Therefore, it is highly probable that men need a higher quantity of formal and informal knowledge in order to invest in the improvement and maintenance of oral health. The results are similar to other scientific studies [9,48] that show that men have a lower level of literacy, while women have more health-seeking behaviours and are more involved in health-sphere activities, spend more time with doctors, and have more medical appointments [9,48].

The present study has its *limitations*. The results are limited by the inherent biases associated with the data of the self-reporting scales, including the bias of social desirability. Another limitation is that the sample includes a high number of individuals with higher education. Therefore, the obtained results cannot be generalized, and further scientific research is needed in this domain. Additionally, longitudinal studies starting from early studies are needed. Another limitation derives from the fact that the data collection provided only the biological gender and not the self-identified gender.

The findings of this study can contribute to a better understanding of the gender differences in oral health attitudes, values, behaviours, and literacy. By considering what the two genders value could lead to an increased awareness of some gender distinctions—this would make decision-making factors require a lot of effort, and resources to be used in a more efficient manner. The study confirms that in the case of men, there is a need for formal education and oral health literacy to influence their oral health behaviour and orientation towards professional services. As we showed in the first part of the study, men use professional services less frequently than women. Therefore, the reduced contact with a dental professional can explain the reduced literacy, which does not allow the acquisition of developed knowledge and positive attitudes regarding the behaviours recommended to promote oral health. Additionally, men are less concerned about oral health and seek less medical information. In this sense, further studies must consider an equivalent constitution of samples of men from different perspectives; for example, socio-professional status, level of education, and urban or rural origin. At the same time, the level of literacy could be correlated with the number of hours worked by men and women, to see if the lack of time is a reason not to use professional services or if it is just dental indifference. Last, but not least, future studies should use extended scales to evaluate oral health literacy, such as Rapid Estimation of Adult Literacy in Dentistry (REALD−30) [49], or Test of Functional Health Literacy in Dentistry (ToFHLiD) [50], together with an oral clinical status assessment.

The results of this study could be used as antecedents for future studies that should analyse the roles of other types of causes that may account for gender psychology in oral health. In addition, the results suggest there is a need to develop dental service strategies in order to approach these differences, and to understand that there is a host of values, attitudes, oral health literacy, and formal education underlying the pattern of dental visits.

It is recommended that medical services should meet the patient’s educational needs in order to facilitate the understanding of medical information. This requires efficient communication and a trusting relationship between the doctor and the patient. We need to assess the extent to which the patient understands the sanogenic message in order to increase the degree of awareness of one’s own health condition, to make patients independent and motivated in their self-practice, and to increase their personal autonomy.

## Figures and Tables

**Table 1:** The socio-demographic characteristics of the sample.

Variables		Males (N = 250)	Females (N = 350)
Age	Mean_age_ (S.D.)	31.79 (14.11)	29.51 (14.47)
Domicile	Urban Rural	84.50% 15.50%	85.10% 14.90%
Education	Primary studies Secondary studies University studies Post-university studies	4% 26.30% 47.00% 22.70%	3% 16.10% 57.22% 23.68%
Work sector	Public Private Unemployed	44.60% 47.60% 7.80%	50.80% 44.80% 4.40%

**Table 2:** The descriptive statistics of the variables involved in research (means, S.D., skewness, kurtosis, consistency coefficients).

Variables	M	S.D.	Min–Max	α	ω	Skew.	Kurt.
**R-DBS**	55.38	23.57	28–139	0.96	0.96	1.02	0.40
Professionalism	22.56	9.56	11–55	0.91	0.92	0.85	−0.34
Comfort	17.17	8.41	9–45	0.93	0.93	1.13	0.64
Lack of control	15.63	7.38	8–40	0.90	0.90	1.07	0.59
**OHVS**	48.10	6.95	19–60	0.77	0.78	−0.73	1.03
Professional dental care	11.77	2.66	3–15	0.55	0.56	−0.64	−0.00
Appearance	13.85	1.81	3–15	0.72	0.73	−1.93	4.36
Flossing	9.02	3.33	3–15	0.79	0.80	0.06	−0.76
Retention of teeth	13.44	1.43	3–15	0.46	0.45	−1.67	4.07
**DNS**	23.81	2.73	7–30	0.71	0.71	−1.12	3.15
**OHLS**	12.62	2.13	5–15	0.63	0.62	−0.82	0.14

Note: R-DBS = dental beliefs survey—revised; OHVS = oral health values scale; DNS = dental neglect scale; OHLS = oral health literacy scale; M = mean; S.D. = standard deviation; skew. = skewness; kurt. = kurtosis.

**Table 3:** Gender differences in oral health-related factors.

Variables		Mean Rank		Mann–Whitney U	Z *
		Males	Females		
**R-DBS**	Professionalism	339.89	271.23	33,661.00	−4.78
	Comfort	339.74	271.34	33,699.00	−4.79
	Lack of control	329.11	279.01	33,368.50	−3.50
	DBS total score	340.10	271.08	33,608.50	−4.81
**OHVS**	Professional dental care	255.60	331.98	32,545.50	−5.37
	Appearance	237.64	344.98	28,020.50	−8.39
	Flossing	241.58	342.13	29,011.50	−7.05
	Retention of teeth	264.57	325.85	34,711.00	−4.42
	OHVS total score	228.29	351.79	25,652.00	−8.63
**DNS**	Dental neglect total score	263.20	326.54	34,438.00	−4.46
**OHLS**	Oral health literacy	223.91	354.88	24,576.00	−9.27

Note: R-DBS = dental beliefs survey—revised; OHVS = oral health values scale; DNS = dental neglect scale; OHLS = oral health literacy scale; * all at the *p* < 0.01.

**Table 4:** Gender differences in terms of items.

Items	Z *
I believe dentists say/do things to withhold information from me *^1^	−6.21
I am concerned that dentists provide all the information I need to make good decisions *^1^	−4.86
Dental professionals say things to make me feel guilty about the way I care for my teeth *^1^	−4.52
Dentists focus too much on getting the job done and not enough on the patient’s comfort *^1^	−4.29
I am concerned that the dentist will do what he wants and not really listen to me while I’m in the chair *^1^	−3.66
My smile is an important part of my appearance *^2^	−6.54
Flossing my teeth every day is a high priority for me *^2^	−7.50
I think it is important that my teeth and gums are a source of pride *^2^	−7.13
The condition of my teeth and gums is an important part of my overall health *^2^	−6.38
I receive the dental care I should *^3^	−4.51
I consider my dental health to be important *^3^	−6.13
How sure are you that you can fill in the personal data from the medical record, the informed consent and the general health assessment form yourself *^4^	−5.58

Note: *^1^—items from R-DBS = dental beliefs survey—revised; *^2^—items from OHVS = oral health values scale; *^3^—items from DNS = dental neglect scale; *^4^—item from OHLS = oral health literacy scale. * all at the *p* < 0.01.

**Table 5:** The inference of the effects of knowledge on values, attitudes, and behaviours on oral health; male sample (N = 250).

Effects Inference	β	SE	t-Value	*p* (2-Sided)
OHL → distrust	−0.46	0.05	−8.61	0.000
OHL →values	0.35	0.05	6.22	0.000
OHL → self-care	0.30	0.05	5.04	0.000
EL → distrust	0.05	0.06	0.84	0.398
EL → values	0.18	0.05	3.00	0.004
El → self-care	0.21	0.05	3.74	0.000

Note: OHL = oral health literacy; EL = education level.

**Table 6:** The inference of the effects of knowledge on values, attitudes, and behaviours regarding oral health; female sample (N = 350).

Effects Inference	β	SE	t-Value	*p* (2-Sided)
OHL →distrust	−0.38	0.04	−8.13	0.000
OHL → values	0.28	0.04	6.64	0.000
OHL → self-care	0.29	0.05	5.02	0.000
EL → distrust	0.03	0.05	−0.54	0.588
EL → values	0.07	0.05	1.29	0.194
El → self-care	−0.01	0.04	0.13	0.891

Note: OHL = oral health literacy; EL = education level.

## Data Availability

The data presented in this study are available from the corresponding authors upon reasonable request.

## Appendix A

**Table A1:** The least different items between males and females/the items with the smallest differences between the two genders.

Items	Z *
When a dentist seems in a hurry, I worry that I’m not getting good care *^1^	−2.02
Dentists don’t seem to notice that patients sometimes need a rest *^1^	−2.20
If I were to indicate that it hurts, I think that the dentist would be reluctant to stop and try to correct the problem *^1^	−2.90
I have had dentists not believe me when I said I felt pain *^1^	−2.30
Dentists often seem in a hurry, so I feel rushed *^1^	−2.80
Being overwhelmed by the amount of work needed (all the bad news) could be enough to keep me from beginning or completing treatment *^1^	−2.90
From my point of view, regular dental care does not justify the cost *^2^	−1.95
I control snacking between meals as well as I should *^3^	−2.43

Note: *^1^—items from R-DBS = dental beliefs survey—revised; *^2^—items from OHVS = oral health values scale; *^3^—items from DNS = dental neglect scale. ***** *p* between 0.05–0.09.
